# Restaurant outlet density and the healthfulness of food purchases: evidence from FoodAPS

**DOI:** 10.3389/fnut.2024.1369240

**Published:** 2024-04-18

**Authors:** Richard Volpe, Xiaowei Cai, Marilyn Tseng, Wilson Sinclair

**Affiliations:** ^1^California Polytechnic State University, San Luis Obispo, CA, United States; ^2^Economic Research Service, United States Department of Agriculture (USDA), Washington, DC, United States

**Keywords:** FoodAPS, National Household Food Acquisition and Purchase Survey, HEI, Healthy Eating Index, food environment, FAFH, food away from home

## Abstract

**Introduction:**

The average American household’s diet and food purchasing patterns are out of sync with federal recommendations. Researchers have connected this with the large and growing rates of obesity, diabetes, and other diet-related ailments in the U.S. Restaurant food has been discussed a potential contributor to unhealthful diets, as it is often calorically dense. We investigate the association between household access to restaurants and diet quality using USDA FoodAPS data and NPD ReCount data.

**Methods:**

We define radii around households to measure restaurant outlet counts and apply a regression analysis incorporating household characteristics.

**Results:**

We find that neither restaurant counts nor openings share many statistically or economically significant associations with average dietary quality. Household characteristics and demographics are far more powerful in explaining variation in diet quality.

**Discussion:**

Our findings align with the large and growing body of empirical research that suggests that personal preferences and other household characteristics are more important than the food environment in explaining food choices and diet quality. Given the extant research on the importance of access to large supermarkets, our results suggest that access to food retailers is more important in explaining diet quality than access to restaurants.

## Introduction

Acquiring healthful and affordable food is challenging for many U.S. households, as 40 percent of individuals in the U.S. lived more than one mile from a food store as of 2015 (1). For a variety of reasons that are not fully understood, Americans’ food purchase and consumption patterns are out of sync with recommendations (USDA-FNS, 2019), and this is associated with a wide range of adverse health effects, including obesity and diabetes (2). The food environment, defined as the number and type of food outlets accessible to households, has been established as a determinant of consumer demand for foods (3). We examine how dietary quality, as proxied by food choices, is associated with the presence of food away from home (FAFH) outlets, including full-service and quick-service restaurants. The access to FAFH outlets and the association between restaurant availability and food choices is of specific policy interest, given their widespread distribution and calorically dense food and beverage offerings. We seek to understand the extent to which the FAFH environment is associated with the healthfulness of food purchases.

The economic, health, and epidemiological literature is replete with studies on the association between the food environment and dietary quality, but relatively little is known about the effects of FAFH access, relative to the FAH environment. Research on FAFH outlet density, which is typically defined as counts *per capita* or counts within defined geographic bounds and is a traditional measure of access to these outlets, and diet quality has reached a near consensus showing that food consumed away from home is typically less consistent with dietary recommendations. Mancino et al. (4) and Todd et al. (5) used the National Health and Nutrition Examination Survey (NHANES) data to show that FAFH, as a share of caloric intake, is associated with decreased dietary quality among adults. Wolfson and Bleich (6) used NHANES to show that the frequency of cooking and eating at home is associated with improved dietary quality and weight loss. Altman et al. (7) studied a sample of overweight children over time and found that reducing FAFH intake improved dietary quality and bodyweight. Despite this body of evidence, the extent to which food outlet density and associated changes in the composition of the local food environment is associated with the healthfulness of food purchases locally is unexplored.

The relationship between food environments and food purchases is not always clear, and empirical efforts to identify linkages in this context are fraught with challenges. The extant research on the links between the local food environment on food choices, dietary quality, and health outcomes has mostly yielded minor impacts. Food choices are potentially endogenous to the local food environment, as individuals can choose where to live based on the food environment they desire, among other factors (8). Unobservable variation across households with respect to attitudes toward healthy foods and nutrition and health education plays a major role in consumer valuation, shopping decisions and dietary quality (9) and render estimates in food environment studies small and insignificant, even in meta-analyses and surveys of the literature (10). Perhaps not surprisingly, studies on the food environment and food access often find counterintuitive associations with the quality of food purchases. Each of the factors and challenges need to be considered when studying the associations between FAFH outlets and the extent to which food choices correspond to federal dietary recommendations.

Quick-service restaurants, also known as fast-food, have attracted considerable attention in the health, nutrition, and epidemiological literature, largely due to their calorically-dense food and beverage offerings and low prices, which may have implications for food choices. Jaworowska et al. (11) surveyed the health and nutrition literature and discussed the various studies that have linked fast-food outlets to obesity, diabetes, and heart disease, particularly among children and low-income populations. Davis and Carpenter (12) and Currie et al. (13) found that household proximity to fast food restaurants, based on straight-line distances, was associated with a significant increase in the risk of childhood obesity. An (14) used national dietary recall data for over 18,000 adults to show that the impacts of fast-food consumption on the total intake of calories, saturated fat, cholesterol, and sodium exceeding recommendations were greater than those of dining in full-service restaurants. Finally, Cooksey-Stowers et al. (15) showed that areas with high concentrations of fast-food restaurants are more effective at predicting US adult obesity rates than are areas of limited food access, which speaks to an association between fast-food patronage and diet quality. Regulations to prevent or limit quick-service entry have been discussed and implemented throughout the U.S. Usually such bans are proposed and/or implemented in the name of improving the local food environment for Americans. Nixon et al. (16) document 77 instances of fast-food zoning bans in the U.S. between 2000 and 2013. Therefore, an improved understanding of the association between local dietary quality and quick-service entry and density has the potential to inform policy on these outlets.

We created a novel dataset to measure the association between the health quality of food purchases with changes in the local food environment, considering FAFH outlets. Associations were measured for changes in the food environment within each of the previous one to five years and within two, three, nine, and thirteen miles of the respondents’ homes. USDA’s 2010 Healthy Eating Index (HEI-2010), which assesses how well a diet aligns with recommendations of the Dietary Guidelines for Americans (DGA), is the benchmark for the healthfulness of diets used in this study. We find predominantly null effects, and we contribute to the body of knowledge suggesting that regulations seeking to limit the entry and proliferation of FAFH outlets may not be effective at addressing dietary quality and related health outcomes in the U.S.

## Data and methodology

There are four datasets used in our study. Our empirical model uses the USDA’s National Household Food Acquisition and Purchase Survey (FoodAPS) for spending behavior, the Healthy Eating Index for the healthfulness of food purchases, and ReCount for the food environment. We also use data from the USDA Food Environment Atlas for model validation. Food choices are recorded and categorized using household-level survey data. Changes in the food environment are determined by examining the change in density of the primary types of FAFH outlets within specified radii of the respondents’ household in each year from 2007 to 2012. Finally, dietary quality is assessed using the HEI-2010.

### FoodAPS Data

FoodAPS is a nationally representative survey of U.S. households to collect comprehensive food purchase data conducted in 2012–2013 and improves upon the limitations of point-of-sale scanner data in two crucial ways for this analysis. First, FoodAPS provides exact geographic coordinates for respondent households, enabling researchers to obtain a clear picture of the household’s food environment by measuring the distance to certain types of food retailers, as opposed to using zip codes or counties as a household’s location. Second, FoodAPS oversamples low-income households, which are often underrepresented in scanner data. Low-income households, those with incomes less than 185 percent of the poverty line, are well-represented in the FoodAPS sample, whereas this group is among the most likely to underreport their food purchases in household scanner data (17) and least likely to meet the DGA (18). The FoodAPS sample is much smaller than the sample used for point-of-sale data, and we explore the notion that FoodAPS households do not necessarily represent geographic areas in terms of demographics or food choices with national USDA data.

The FoodAPS survey was the first to collect comprehensive data regarding food spending behavior from food retailers such as grocery stores as well as the distance traveled and method of transportation to make the purchases (19). The FoodAPS survey was conducted between April 2012 and January 2013 and includes 4,826 households. Through the survey, respondents recorded their complete food acquisitions (including quantities, prices, and outlet type) for a one-week period as well as information regarding the sociodemographic attributes, food shopping habits, health awareness, and economic well-being of the household (20).

Given these improvements, FoodAPS has been utilized to identify meaningful insights regarding public health and food environments. For instance, Rahkovsky and Snyder (21), found a relatively modest negative correlation between low-income, low-access areas and healthful food purchases, a correlation that is higher among urban residents than among non-urban residents. Furthermore, there is evidence that households in low-income, low access neighborhoods purchase the majority of their unhealthful foods at supermarkets that also supplied healthier options (22), and households participating in the Supplemental Nutrition Assistance Program (SNAP) only purchased slightly more of their food at convenience stores compared to non-SNAP participants (23).

**Figure 1:**
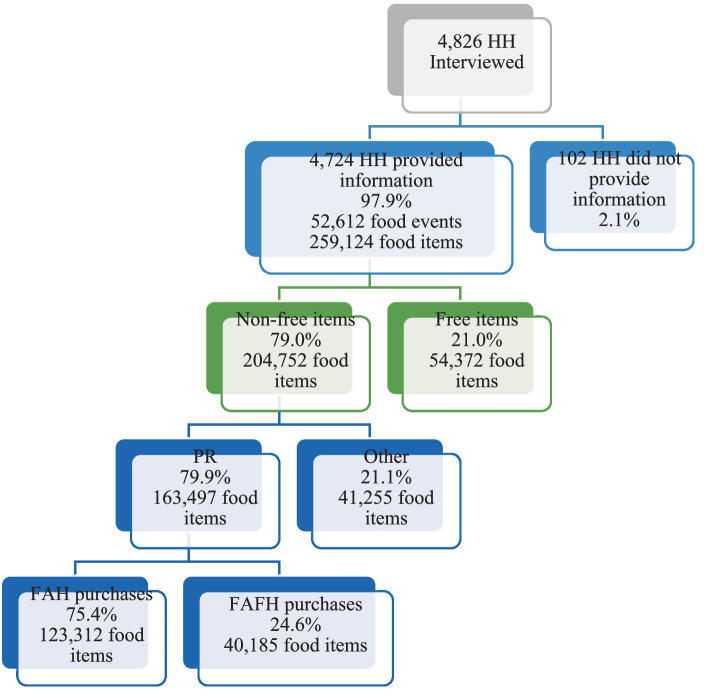
Numbers of FoodAPS participants in sample subset. HH, household; FAH, food-at-home; FAFH, food-away-from-home; PR, primary respondent. Source: Author’s calculations using 2012–2013 data from FoodAPS.

Almost all households reported acquiring or purchasing food at least once during the week (98 percent or 4,724 households). In total, there were 52,612 food events where a household member purchased or acquired at least one food item, with a total of 259,124 items acquired or purchased with nutrition information. Because we focus on the relationship between changes in the food environment and the nutritional quality of foods acquired, we limit the analysis to food purchases and exclude food items that were acquired for free, e.g., donations, gifts, or work functions. Free food items not considered in the analysis account for 21 percent of all food items reported. Furthermore, we focus on foods purchased by the primary shopper only, as this person is likely to have the most influence on the overall nutritional quality of the household’s dietary patterns. In FoodAPS, each household’s main food shopper or meal planner served as the household’s primary respondent (PR). Compared to other household members, purchases made by PRs made up the bulk of the food items over the recall week (80 percent of all non-free food items). Finally, FoodAPS captures FAH as well as FAFH purchases. About three-quarters of all the items purchased by the PR were purchased for FAH consumption and one-quarter were purchased for FAFH (Figure 1).

The average age of PRs in the subsample is 50 years old, 69 percent were females, and 46 percent were married. One-third of the PRs report their highest level of educational attainment to be a high school diploma or GED or less, one-third some college, and one-third a bachelor’s degree or more. Overall, 54 percent of the participants are employed. Thirteen percent report being Hispanic, 71 percent were non-Hispanic and White, and 11 percent report being non-Hispanic and Black (Table 1).

**Table 1:** Summary statistics for FoodAPS primary respondents.

Item	Mean	Std. Err.	[95% coef. Interval]	
Age (years)	50.32	0.543	49.21	51.44
Female (percentage)	68.92	1.480	65.88	71.96
Married (percentage)	45.60	1.543	42.43	48.77
Employed (percentage)	54.17	1.384	51.32	57.01
High school diploma/GED or less (percentage)	33.41	2.084	29.13	37.69
Some college (percentage)	33.26	2.606	27.91	38.62
Bachelor’s degree (percentage) or above	33.33	2.637	27.91	38.75
Hispanic (percentage)	12.71	0.304	12.08	13.33
Non-Hispanic White (percentage)	71.24	0.814	69.57	72.92
Non-Hispanic Black (percentage)	11.06	0.576	9.88	12.25
Normal weight (percentage)	35.45	1.795	31.76	39.14
Overweight (percentage)	32.44	1.227	29.92	34.96
Obese (percentage)	32.11	1.838	28.33	35.89
Household size (number)	2.43	0.02	2.39	2.47
Food insecure (percentage)	14.86	1.099	12.60	17.12
SNAP participant (percentage)	13.19	0.259	12.66	13.73
Non-SNAP participant high-income (percentage)	17.28	1.119	14.98	19.58
Non-SNAP participant low-income (percentage)	69.52	1.118	67.22	71.82

*N* = 4,152; GED, General Equivalency Diploma; SNAP, Supplemental Nutrition Assistance Program. Source: Calculations using 2012–2013 data from FoodAPS. Survey weights were used to compute national representative estimates.

### ReCount data

Outlets for FAFH are identified and counted using NPD ReCount. ReCount has restaurant data for the U.S., which is disaggregated into Full-Service Restaurants (FSR) and Quick-Service Restaurants (QSR), which is an important distinction for this analysis. Eating away from home often means less healthful purchases, particularly in the case of QSRs or fast-food (24–26). We use both formats to define and measure FAFH outlet density in our empirical analysis. The ReCount data used in this analysis range from 2007 to 2012 and describe specific location (i.e., address), type of restaurant, and changes in density within a specific distance of each respondent.

### 2010 Healthy Eating Index

The healthfulness of food purchases reported in FoodAPS is measured using the 2010 Healthy Eating Index (HEI-2010). The FoodAPS data include food acquired for free by households, but we calculate the HEI-2010 using only foods purchased from FAH and FAFH outlets, as we are interested in how changes in the local food environment are associated with consumer behavior, as it can be observed by industry practitioners.

The USDA’s Center for Nutrition Policy and Promotion and the National Cancer Institute developed the HEI-2010 to assess compliance with the Dietary Guidelines for Americans, 2010 (2, 27). The HEI-2010 scores across nine adequacy components (i.e., food groups that individuals should consume a certain amount of daily) and three moderation aspects (i.e., food groups that should be limited) for a maximum total score of 100 across the 12 components (Table 2). The HEI-2010 has been used to assess a wide range of food sets such as a community’s environment or menu offerings (28) as well as to analyze the diet quality of the complete U.S. population and subpopulations across food environments (29). The average HEI-2010 score for PRs in this analysis is 51.8, lower than the national average of 59 found in a 2013–2014 survey (30), which is consistent with a sample heavily weighted toward lower-income households.

**Table 2:** Healthy Eating Index-2010 components and their scoring standards.

Component	Maximum points	Standard for maximum score	Standard for minimum score of zero
Total vegetables	5	≥1.1 cup equiv. per 1,000 kcal	No vegetables
Total fruit	5	≥0.8 cup equiv. per 1,000 kcal	No fruit
Whole fruit	5	≥0.4 cup equiv. per 1,000 kcal	No whole fruit
Greens and beans	5	≥0.2 cup equiv. per 1,000 kcal	No dark green vegetables or beans or peas
Whole grains	10	≥1.5 oz. equiv. per 1,000 kcal	No whole grains
Dairy	10	≥1.3 cup equiv. per 1,000 kcal	No dairy
Total protein foods	5	≥2.5 oz. equiv. per 1,000 kcal	No protein foods
Seafood and plant proteins	5	≥0.8 oz. equiv. per 1,000 kcal	No seafood or plan proteins
Fatty acids	10	(PUFAs + MUFAs)/SFAs ≥2.5	(PUFAs + MUFAs)/SFAs ≤1.2
Moderation			
Refined grains	10	≤1.8 oz. equiv. per 1,000 kcal	≥4.3 oz. equiv. per 1,000 kcal
Sodium	10	≤1.1 gram per 1,000 kcal	≥2.0 grams per 1,000 kcal
Empty Calories	20	≤19% of energy	≥50% of energy

Kcal, Kilocalorie, a measure of energy; MUFA, monounsaturated fatty acid; PUFA, polyunsaturated fatty acid; SFA, Saturated fatty acid. Source: Author’s using data from the National Institutes of Health’s Healthy Eating Index website.

### USDA Food Environment Atlas

The USDA Food Environment Atlas is a publicly available tool that allows users to visualize county- or state-level data on food outlets, demographics, food assistance benefits, food price indicators, and other community characteristics. The underlying data for the Atlas was constructed from a combination of publicly available federal datasets, e.g., the American Community Survey, and proprietary sources, e.g., Nielsen TDLinx. The complete details on the dataset are available at ERS (31).

The comprehensive national scope of the Atlas is ideal for model validation in our case, as we draw conclusions on consumer behavior based on a limited sample of households. The Atlas data are updated by USDA-ERS periodically, but we rely exclusively on the 2015 edition, which includes outlet and demographic data from 2012, which is the best match for the FoodAPS data.

### Econometric model

This study aims to understand how entries of FAFH formats are associated with adherence to dietary recommendations using an ordinary least squares regression (OLS) framework. This method does not allow us to infer causality but does provide us with associations that help understand public health across changing food environments. Respondents’ HEI scores are modeled as a function of a vector of food environment variables while controlling for household characteristics. The food environment variables included in the analysis represent recent changes in the number of FAFH establishments across various distance thresholds.

We aggregated the total number of quick-service and full-service restaurants within 0.5, 1, 2, and 5 miles by household for each year, utilizing the NPD ReCount data from 2007 to 2012, spanning a total of 5 years. We selected this five-year timeframe because the FoodAPS data are from 2012, and we aim to capture the impact of long-term changes prior to that in households’ local FAFH environment on their HEI scores.

Our estimation strategy relies on the assumption that FoodAPS households are representative of other households nearby, with comparable demographics. We also assume that mobility among FoodAPS households is minimal during this five-year span. Using data from 2010–2011, Mateyka (32) showed that annual mobility among US households was 9.7% and was lowest among low-income households, supporting this notion and speaking to the concern that low-income and other disadvantaged populations sampled by FoodAPS are affected by food environments they did not select. Our specification for household *i* is given by:


(1) $$\mathrm{HE}I_{i}=\beta_{0}+\beta_{1}x_{\mathrm{irdt}}+\beta_{2}b_{ird2012}+\bar{\gamma }\bar{Z_{i}}+\epsilon_{i}$$

where *x* is the change in the number of establishments around respondent *i*, in the last *t* years, for the distance threshold *d*, and the type of restaurant *r* (FSR or QSR) and *b* is the number of restaurants *r* around respondent *i*, in 2012, within distance threshold *d*. Z is a vector of control variables, including gender, age, marital status, employment, education attainment, rural vs. urban household, ethnicity, health status, household size, food security status, and SNAP participation. Additionally, we include a dummy variable indicating non-SNAP households categorized as low-income, to ensure that we are capturing the income effect. Definitions and descriptive statistics for these variables are provided in Table 1. Finally, ϵ represents cluster errors at the primary sampling unit. $\beta_{2}$ is our primary coefficient of interest and represents the change in HEI-2010 scores when the number of establishments of restaurant type *r* in the last *t* years and within distance threshold *d* changes.

As noted above, studies on the impacts of the food environment are subject to endogeneity concerns. We do not claim causality with respect to impact on diet quality, as we have only one year of purchase data. However, given that we measure marginal changes to the food environment that are observed by static households, we argue that the identification strategy captures the impact changes in FAFH outlet density, rather than household choices. Moreover, we used data from the USDA Food Environmental Atlas to investigate correlations among restaurant outlets *per capita* and demographic measures that align with those in FoodAPS, including income, age, race, and ethnicity. Supporting our approach, the correlations are small in magnitude, as the largest is 0.30. Additionally, the signs of the correlations do not conform broadly to expectations drawn from theory and evidence. For example, the correlation between median household income and fast-food restaurant density is positive. These results are available from the authors upon request.

## Results

**Figure 2:**
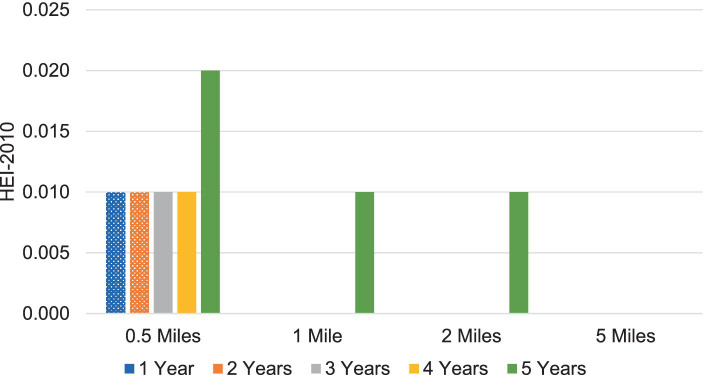
Significant associations between FAFH 2012 store counts and HEI-2010 scores. Each bar represents regression coefficient ( $\beta_{2}$ ) from Eq. 1 within *t* years and *d* miles. The displayed values are statistically significant at the 0.1 percent level. Calculations using 2012–13 data from FoodAPS and National Institutes of Health’s 2010 Healthy Eating Index website.

The results of estimating Eq. (1) link HEI scores to FAFH counts and entries. The complete set of results, including the estimated coefficients for all control variables, is available from the authors upon request. Figure 2 illustrates the magnitude of β_2_ in Eq. (1) for FAFH. It is readily apparent that the associations between outlet density, measured here as counts within fixed radii, and HEI scores are weak for FAFH. Most coefficients are statistically zero, and outlet density is associated with HEI increases of about 0.01 for one to four years, jumping to 0.02 for five years within a half mile. We also find evidence of weak associations at the five-year mark at one and two miles. As displayed in this figure, the FAFH stores counts across all years significantly affect household HEI scores within a 0.5-mile radius. However, as the distance from FAFH outlets increases, the impact of the number of nearby restaurants on household HEI scores diminishes.

Most of the *β_2_* estimates are largely insignificant; we find limited evidence for associations between FAFH density and HEI scores. Table 3 reports the estimated differences between HEI scores based on FAFH density change and the average HEI score in our sample. For all FAFH density changes, the differences are small in magnitude, and mostly negative. For example, the presence of one additional FAFH store within 0.5 miles over three years is associated with a 0.3% decrease in the HEI score compared to the sample average. There are some significant differences at three, four, and five years within two miles or less, but all are −0.3% or smaller. Additionally, we do not observe qualitative differences between quick-service and full-service restaurant density changes. Both outlets generally mirror the overall FAFH results, showing small, negative, and mostly insignificant differences.

**Table 3:** Relative difference in HEI-2010 scores compared to sample average, density changes of FAFH.

All FAFH density changes				
	0.5 miles	1 mile	2 miles	5 miles
1 year	0.4%	0.0%	0.0%	0.0%
2 years	0.0%	−0.1%	0.0%	0.0%
3 years	−0.3%*	−0.1%	−0.1%**	−0.0%**
4 years	−0.2%	−0.1%*	−0.1%***	−0.0%***
5 years	−0.2%	−0.2%**	−0.1%***	−0.0%***
Full-service restaurant density changes				
	0.5 miles	1 mile	2 miles	5 miles
1 year	0.2%	0.1%	0.0%	0.0%
2 years	−0.5%	−0.1%	0.1%	0.0%
3 years	−0.6%**	−0.2%	−0.1%*	−0.0%*
4 years	−0.5%	−0.2%	−0.2%**	0.0%
5 years	−0.4%	−0.2%*	−0.2%***	−0.0%***
Quick-service restaurant density changes				
	0.5 miles	1 mile	2 miles	5 miles
1 year	0.8%	0.0%	0.0%	0.0%
2 years	0.3%	−0.1%	0.0%	−0.1%
3 years	−0.1%	−0.1%	−0.1%**	−0.1%***
4 years	0.1%	−0.2%	−0.2%***	−0.1%***
5 years	0.1%	−0.2%*	−0.2%***	−0.1%***

***Differences are calculated based on coefficients that are significant at the 0.01 level. **At the 0.05 level. *At the 0.10 Level. Values represent the percentage difference between the HEI-2010 scores for individuals within t years and d miles and for each r store type as compared to the sample average. Source: Calculations using 2012–13 data from FoodAPS and National Institutes of Health’s Healthy Eating Index website.

### Evidence from the Food Environment Atlas

We recognize that our data and empirical approach cannot establish causality. Moreover, while the FoodAPS data are nationally representative, there is little question that in some areas, the estimated associations are driven by a small number of households. This leads to concerns that our findings may not be representative of the actual relationships connecting outlet openings and household behavior in the U.S. To investigate this possibility, we use the data from the 2014 USDA Food Environment Atlas (henceforth the Atlas).^1^ This is a county-level dataset including measures of outlet counts, as well as a vast array of demographic and food environment descriptors. While the Atlas does not measure store openings or closings directly, it does not measure local food choices, and its measurements are restricted to county boundaries, it has the advantage of being nationally comprehensive, with complete and uniform coverage of rural and urban areas.

In using the Atlas, we proxy for all the variables constructed in FoodAPS and used in Eq. (1). To proxy for store openings and closings, we use the changes in outlet counts *per capita*, 2007–2011. These are available for fast-food restaurants and full-service restaurants. There are no measures of food consumption or dietary quality in the Atlas, and we therefore use median household income, the poverty rate, the percent of households in poverty, the adult diabetes rate, the adult obesity rate, and the change in recreational facilities *per capita*, 2007–2011. The correlations, across all US counties, are reported in Table 4.

**Table 4:** Correlation coefficients for store counts and demographics as measured by the USDA Food Environment Atlas.

	% FastFood 07–11	% FullServ 07–11	Med HH Income 2010	Poverty Rate 2010	% in Poverty 2011	% Diabetes 2010	% Obese 2010	% RecFac 07-11
% FastFood 07–11	1.00							
% FullServ 07–11	−0.05	1.00						
Med HH Income 2010	0.01	−0.02	1.00					
Poverty Rate 2010	0.01	0.04**	−0.77***	1.00				
% in Poverty 2011	0.03*	0.05***	−0.41***	0.65***	1.00			
% Diabetes 2010	0.03**	0.06***	−0.55***	0.53***	0.39***	1.00		
% Obese 2010	0.02	0.05***	−0.48***	0.45***	0.34***	0.70	1.00	
% RecFac 07–11	−0.01	0.00	0.04**	−0.04**	−0.01	−0.06***	−0.02	1.00

***Correlation coefficient is statistically significant at the 0.0 l level. **At the 0.05 level. *At the 0.10 level. Authors’ calculations using the 2014 USDA Food Environment Atlas.

In most respects, the correlations corroborate our regression results and findings. The correlations between our diet quality proxies and FAFH outlets are statistically significant, but very small and not economically significant. An increased availability of both FSR and QSR is associated with slightly higher rates of diabetes and obesity. As noted above, we also observe positive correlations with the poverty rate, and inverse correlations with household income and the availability of recreational facilities. The results support the notion that FAFH outlet density is associated with numerous other demographic and market factors that have been shown to be related to poor dietary quality and related health outcomes, which in turn helps to explain why our findings, and those of many related studies, have found limited associations between dietary quality and the food environment.

## Discussion and concluding remarks

We combine the USDA FoodAPS data with NPD ReCount data to estimate associations between changes in local FAFH counts with local diet quality, as measured using HEI scores. We find that neither FAFH counts nor FAFH openings share many statistically significant associations with average dietary quality. We do not find evidence that increased FAFH outlet counts are associated with decreased HEI scores. Moreover, there is little evidence of meaningful associations between FAFH density and diet quality. The largely null associations with FAFH outlets, based on both total store count and changes therein, help inform the ongoing discussions of fast-food chains in the media and in policy circles. Proposed and enacted regulations on fast-food restaurants include, but are not limited to, entry bans (33), requirements to post calorie counts (34), and limitations on ingredients to be used in menu offerings (35). Our study contributes to the ongoing discussion and growing body of evidence by further demonstrating that such implementations are likely to have limited impacts on food choices, given the weak associations found between restaurant density with local diet quality. Rather, consumer education [e.g., (36)] and access to full-sized supermarkets [e.g., (10)] are likely to be more impactful in improving average dietary quality in the U.S.

The positive associations between FAFH counts and HEI scores are perhaps counterintuitive. However, it is worth stressing that these estimates likely reflect the fact that the FAFH counts, and food-at-home store counts can be positively correlated spatially. Moreover, these associations are very small, and we do not ascribe causality to them. Therefore, where there is greater access to restaurants, there is also greater access to supermarkets and supercenters. We investigate the associations between health and FAFH outlets using data from the publicly available USDA Food Environment Atlas and find positive correlations between adverse health outcomes and FAFH density, but the magnitudes are very small.

Our study is not without limitations. The FoodAPS sample, while highly granular in measuring food purchases, does not measure food consumption. The data are also more than a decade old, raising concerns about changes in the foodservice sector since the time of data collection and consumers’ attitudes toward it. COVID-19 is particularly salient, given that consumers were forced to eat predominantly at home, and, at the time of writing, it remains unclear the extent to which food sales away from the home will rebound to pre-pandemic levels. Moreover, we are unable to establish causal, time series impacts based on changes in outlet density since we only have one year of food purchase records. Future research on this topic should identify the impacts of retail outlet density, across formats, using longitudinal data, ideally drawn from the period after the COVID-19 lockdowns. Finally, future research on the determinants of store entry or exit decisions would help inform our understanding of the impacts of outlet density on factors such as diet quality, food access, and food prices.

## Data availability statement

The data analyzed in this study is subject to the following licenses/restrictions: both restricted use FoodAPS and NPD ReCount are proprietary and can only be accessed via USDA-ERS with a third party agreement. The dataset used to conduct this study is stored securely at USDA and can be accessed with permission for replication purposes. Requests to access these datasets should be directed to WS, wilson.sinclair@usda.gov.

## Author contributions

RV: Conceptualization, Funding acquisition, Writing – original draft, Writing – review & editing. XC: Conceptualization, Data curation, Formal analysis, Methodology, Software, Writing – original draft, Writing – review & editing. MT: Data curation, Formal analysis, Methodology, Writing – original draft, Writing – review & editing. WS: Data curation, Funding acquisition, Investigation, Project administration, Resources, Writing – original draft, Writing – review & editing.

## Footnotes

^1^This is an older version of the Atlas that is archived and available via the USDA Economic Research Service. It covers approximately the years of interest for our study.
